# ‘No matter what the cost’: A qualitative study of the financial costs faced by family and whānau caregivers within a palliative care context

**DOI:** 10.1177/0269216315569337

**Published:** 2015-06

**Authors:** Merryn Gott, Ruth Allen, Tess Moeke-Maxwell, Clare Gardiner, Jackie Robinson

**Affiliations:** School of Nursing, The University of Auckland, Auckland, New Zealand

**Keywords:** Economic, cost, financial, palliative, family, indigenous

## Abstract

**Background:**

There has been significant attention paid in recent years to the economic costs of health service provision for people with palliative care needs. However, little is known about the costs incurred by family caregivers who typically provide the bulk of care for people at the end of life.

**Aim:**

To explore the nature and range of financial costs incurred by family caregiving within a palliative care context.

**Design:**

In-depth qualitative interviews were conducted with 30 family/whānau caregivers who were currently caring for someone with a life-limiting illness or had done so within the preceding year. Narrative analysis was used to identify impacts and costs at the personal, interpersonal, sociocultural and structural levels.

**Setting:**

Auckland, New Zealand.

**Findings:**

Costs of caregiving were significant and, for participants, resulted in debt or even bankruptcy. A range of direct (transport, food and medication) and indirect costs (related to employment, cultural needs and own health) were reported. A multi-level qualitative analysis revealed how costs operated at a number of levels (personal, interpersonal, sociocultural and structural). The palliative care context increased costs, as meeting needs were prioritised over cost. In addition, support from statutory service providers to access sources of financial support was limited.

**Conclusion:**

Families incur significant financial costs when caring for someone at the end of life. Research is now needed to quantify the financial contribution of family and whānau caregiving within a palliative care context, particularly given attempts in many countries to shift more palliative care provision into community settings.

**What is already known about the topic?**

- Family members provide the majority of care for people with palliative care needs.
- Research conducted with older people indicates that the financial costs associated with family caregiving can be significant.
- Little is known about the nature and extent of these costs within a palliative care context.

**What this paper adds?**

- This study identifies that family caregivers experience a range of direct and indirect costs associated with caregiving.
- The palliative care context exacerbates many of these costs.
- Support from statutory service providers to access sources of financial support was limited.

**Implications for practice, theory or policy**

- This study indicates an urgent need for policy makers to consider the financial costs of family caregiving, particularly within the context of drives to increase community-based palliative care provision.
- Future research is needed to quantify these costs.
- Culturally appropriate methodological approaches must be used to ensure the inclusion of research participants from indigenous and minority cultural and ethnic groups who are often disproportionately affected by the costs incurred.

## Introduction

A key policy priority in many developed countries is shifting the provision of palliative and end-of-life care from acute hospital settings into the community.^1,2^ One perceived benefit of this approach is a reduction in acute hospital costs for people in the last year of life; it is argued that this money would be better spent providing community health services which could enable people to remain in their own home up until death.^3^ However, one issue that has been neglected within this debate is that reducing hospital admissions would have implications not only for community service provision but also for family caregivers who, it has been estimated, already provide 75%–90% of home-based care for people who are near the end of life.^4^

A recent systematic review conducted to explore the financial costs incurred by family caregivers identified no previous studies focusing specifically upon the financial costs of family caregiving at the end of life, although there was some evidence that these costs were significant.^5^ Indeed, studies were identified which demonstrated that economic costs associated with caregiving have negative implications for the well-being and health of family caregivers,^6,7^ and financial strain is even associated with family preference for comfort care over life-extending treatment.^8^ Since the review was conducted, one article has been published which does quantify the costs of caregiving for family caregivers in Canada. Drawing on data from the family caregivers of patients with cancer accessing a home-based palliative care programme, Chai et al.^9^ concluded that ‘unpaid care costs accounted for the largest portion of total palliative care costs, averaging 76.7% over the last year of life’ (p. 34). This study therefore confirms that, within the Canadian context, family caregivers of people with cancer utilising specialist palliative care services are incurring a far greater proportion of caring costs at the end of life when compared with those incurred by health services. Research is now needed which extends these findings beyond cancer to other diagnostic groups and national contexts.

In the absence of further research, there is a danger that economic evaluations conducted within a palliative care context will only capture those costs which can be easily measured, namely, those incurred by statutory service providers. For example, the recent UK palliative care funding review to inform policy making and commissioning decisions in relation to palliative care focused only upon those costs incurred by the state.^10^ The failure to adopt a societal approach to costing by including family caregiving costs runs the risk of decision-making at a policy level which shifts even more of the costs of caregiving onto families, with significant implications for both their well-being and their capacity to care.

## Aim

To explore family and whānau carers’ experiences of the financial impact of caring within a palliative care context (whānau is most often translated as ‘family’, but its meaning also encompasses physical, emotional and spiritual dimensions (http://www.teara.govt.nz/en/whanau-maori-and-family/page-1)).

## Methods

The choice of qualitative methods was guided by the research question^11^ and the exploratory nature of the study. Our approach was iterative and multi-disciplinary, within the traditions of narrative inquiry.^12–14^ Semi-structured interviews were held with 30 caregivers who were either currently caring for a person with palliative care needs or had done so in the past year. Following approval from the University of Auckland Research Ethics Committee, 17 participants (14 non-Māori and 3 Māori) were recruited through a tertiary hospital palliative care service. Clinical staff identified eligible carers and invited them to talk to a research nurse if interested in participating. Only three of the carers approached declined participation in the study, all for reasons unconnected with the research itself. A second recruitment strategy was adopted to ensure appropriate representation of Māori caregivers. A total of 13 participants (9 Māori and 4 non-Māori) were recruited via community newspaper articles about the research and Māori radio and TV coverage. Māori, the indigenous people of Aotearoa, New Zealand, tend to have poorer health than non-Māori and a higher level of unmet need for health care generally.^15^ Māori who live in more deprived areas are more likely to have greater unmet primary health care needs and therefore face a potentially higher cost burden relative to more affluent carers, so were an important target group for this research.^15^ Previous research has indicated that targeting community media is a successful strategy for recruiting Māori, who are under-represented not only in research^16^ but also as users of specialist palliative care services.^17^ Sampling was purposive to include a mix of information-rich participants with diverse demographic characteristics (age, gender, ethnicity) and involved in various types of carer relationships.^18^ Māori were over-sampled as they comprise 14.9% of the population but are over-represented in health care inequity. Sufficient diversity in terms of these characteristics was believed to have been achieved when we had recruited 30 participants, which is also considered an adequate sample size for an in-depth, exploratory study of this type.^18^ Interviews were conducted by T.M.M. (Māori participants) and R.A. (non-Māori participants). Participants were recruited and interviewed over 6 months from November 2012. All participants provided written informed consent prior to interview. Participant details are summarised in Table 1.

**Table 1.** Participant characteristics (*n* = 30).

Characteristic	*n*
Age range of caregivers	22–79 years
Gender	
Male	7
Female	23
Self-identified ethnicity	
Māori	12
European New Zealander	13
Other (Cook Island, Samoan, Tongan)	5
Caring for	
Mother	14
Father	5
Spouse/partner	5
Other relative (e.g. sibling, great-uncle)	4
Friend/client	2
Life-limiting illness (primary)^a^	
Type 1 (cancer)	19
Type 2 (COPD, cardiac, diabetes)	7
Type 3 (dementia, motor neuron)	4
Length of time caring^b^	
Less than 1 month	2
1–6 months	6
>6 month–1 year	4
>1–2 years	8
>2–4 years	6
More than 4 years	4
Household income^c^	
<NZ$50,000 (low)	17
NZ$50,001–NZ$100,000 (medium)	10
>NZ$100,000 (high)	2

COPD: chronic obstructive pulmonary disease.

a Illness trajectory types are as follows: (1) short period of evident decline, (2) chronic with acute episodes and (3) prolonged dwindling.^19^

b Participants variously defined period of care; end-stage ‘palliative’ may be just weeks but full-time provision of care in context of life-limiting illness may have been years.

c *N* = 29, as the family of one care recipient who was in a long-term residential care facility did not wish to disclose their income. Household income could include carer’s income, care recipient’s income (if living in household) and income from other family members residing in household (e.g. spouse) and does not reflect costs, such as how many children supported on that income and accommodation costs. The mean household income in 2012 in New Zealand was NZ$81,067 (mean individual income NZ$38,843).

Interviews were conducted at participants’ homes or a relative’s home (*n* = 25) by telephone (*n* = 2), at the hospital (*n* = 1) or in a café (*n* = 2) depending on participant preference. Interviews lasted between 30 and 90 min. All interviews conducted by the Māori researcher incorporated a Kaupapa Māori approach. Kanohi-ki-te-kanohi (face-to-face) interviews catered for the rangatira (chiefly status) of participants and Māori cultural research protocols were observed.^20^

The interview guide was informed by relevant literature^5^ and covered the following key areas: experiences of caring, financial costs in relation to both day-to-day care and emergency situations, who else was involved in caring and related costs and whether financial assistance had been received from elsewhere (family, loans, credit, insurance, state support). Views were also sought about appropriate methods for conducting research in this area; these data will be reported elsewhere. Interviews were digitally recorded with participants’ consent and transcribed verbatim. Summaries of interview data were presented back to participants for their feedback to maximise methodological rigour and, in particular, the confirmability of findings.^21^

Qualitative data software (NVivo 10) was used as the filing system for the initial categorising and overview of responses to interview questions, experience-centred narratives,^13^ cost areas and cultural concerns. A more focused analysis was then conducted using the narrative gerontology framework of personal, interpersonal, sociocultural and structural dimensions of experience.^22^ Analysis was led by T.M.M. (Māori) and R.A. (non-Māori); a selection of transcripts was independently reviewed by C.G. and coding was carried out by consensus to ensure rigour and trustworthiness. This framework supported analyses of financial costs which operate at all these levels. Verbatim quotes are presented with anonymised initials and the relationship to the person with life-limiting illness (e.g. partner, daughter).

## Findings

Participants reported a wide range of experiences with relation to the costs of caring and described the significant implications of these costs. Participant data have been summarised under the following themes: the motivations for caring, the range and nature of costs, the impact of the ‘end of life’ phase on costs, the impact on wider family and the lack of support systems. These themes can best be understood within the context of a multi-level narrative gerontology model of personal, interpersonal, sociocultural and structural domains, as will be described below.

While motivations and meanings of care were not the focus of this research, the sensitivities of talking about money ‘at a time like this’ (i.e. when someone had a life-limiting illness) meant participants always framed their talk of costs with comments on the importance and meaning of care to them. As one participant reported,

[Money] never came into the equation … It was a lot of hard work but when I look at it I’m glad I was there and not strangers. (YT, Māori partner)

Māori participants’ caregiving commitment was often informed by cultural values steeped in āroha (compassion) and manaakitanga (preservation of mana and dignity), which were prioritised over care costs. Many participants invoked notions of reciprocity in their discussions:

The way I see it is a parent raises a child, their whole role is to look after the child until they become an adult and then I see it, once you’re an adult we should repay that back. You know, because your parent’s health and everything starts to fail as they get older. So my obligation is to them. (STA, Māori daughter)

However, it was clear that the financial costs incurred by caregiving were significant for all participants, although the extent of the financial impact of caregiving was determined by participants’ current financial situation and therefore varied markedly within our sample. Participants who reported the highest levels of financial resource considered themselves ‘lucky’ and reported only having to cut back on planned expenditure, such as a trip overseas to visit a new grandchild, in order to meet their caring costs. However, others with fewer financial resources reported a range of more serious repercussions. These included incurring significant debt, moving to a smaller house and, in the most extreme cases, going without food because they could not afford to buy enough for everyone in the household:

Sometimes food, the children are fed and we adults just have the leftover, so we could make ends meet. But that’s, but we always think of it’s temporary, and whenever the day will come, it will end. Things will be back to what, to whatever we usually do. (CT, Tongan daughter)

### Range of costs incurred

Participants reported that a broad range of direct and indirect costs were incurred by caregiving (see Table 2).

**Table 2.** Range of costs of family/whānau caregiving.

Transport	I have two sisters who are in Melbourne, who are, they have to travel. So they travel in every alternate week, so it’s like, when it’s their pay day they hop on a plane, come for the weekend. And then they fly back with their children, and then they work for the alternate weekend for the next pay day. (CT, Tongan daughter, caring for mother)^a^
	We’ve had to get an ambulance because she wants it, it’s really uncomfortable for her and so it’s best to have them come out so that she can be rested up in bed and she can lie on her side and … [it’s] $75 per call-out yeah. (KU, Tongan daughter)
Parking	The parking was $18 a day for 7 days a week … and the girls [adult daughters] would come in as well … So I just went in and stayed there all day, I went in first thing in the morning and stayed ‘till 10 o’clock at night. And she was in there probably, three sessions that she spent in there, up to a week. But yeah, that probably was the biggest cost for the whole thing. (CW, NZ European husband)
	It’s a long travel and it’s a long commute for us and it’s the cost really because there’s parking involved too. So sometimes we can get away with one person staying at the Domain [a nearby park] with the car, while the other two visit, but we don’t usually go every day. (YU, Tongan daughter, who is caring for mother, with father and sister KU)
Utility	Power went up because she was home all the time, so the TV was on all the time; she was taking massively long showers when she was initially ill ’cause you know when you’re sick a hot shower does you well. Yeah that was on all the time as well because she was home, to stay warm. So yes our power went from $520 a month down to, after she’d gone, to $220, so it nearly halved. (EW, NZ European husband)
	And the power, yeah it was the power ’cause he got really cold so we had to use the heater like 24/7 we had to leave the heater on and our power bill shot up like a million dollars the last month. (KE, Māori great-niece caring for great-uncle)
Telephone/Internet	Yeah, it was like that can’t use up that much Internet, we’re like, what the hell?! [they had gone way over data cap on account] … it was just searching for things like occupational therapists and what he was entitled to, just the little things that we were always on the Internet for and just trying to figure out how to keep a lung patient comfortable. (KE, Māori great-niece)
Food (see Table 3)	There’s food ’cause she, ’cause mum doesn’t eat, okay? When mum feels like eating, if mum said to me, ‘Oh I feel like scallops’, I’d hunt the earth down to find scallops for mum because if she’s going to eat that I’d rather her have that. (ED, NZ European daughter)
Clothing & bed linen	We don’t want her lying on the same sheet every day, so once she gets a wash, all that gets removed, we place it into the washing machine, wash, dry. (CT, Tongan daughter)
	New pyjama pants and like easy to get on and off clothes. So that would have been about a hundred dollars. (MW, NZ European daughter)
Equipment/house modification	We’re about to spend a thousand dollars on a chair so we can get him in and out. (MS, Cook Island daughter)
	We did buy a wheelchair, which was several hundred dollars but it was just, it was really good at the end when she was, couldn’t hardly walk. (BC, NZ European son)
Alternative or complementary therapies	That was really costly, alternative medication, it was a Chinese herbalist, I think it cost us about $340 or $350 per week for 4 weeks. (MR, Māori daughter caring for father)
Professional carers	Yes, I paid someone … I mean that’s just what you do, it’s no different to having children … I wouldn’t have left her on her own … So I paid one that was about $1,500 but there’s still another couple to come in and then I’ve paid, the cash one must be about $1,000 or something. (GB, NZ European daughter)
Medication	Some of [the medications] are subsidised – I mean you’re not talking about two or three pills, but she was taking a cocktail of drugs for different things and $30/$40 for a whole prescription which doesn’t seem like a lot of money but every week it’s a lot of money when you aren’t working, ’cause you’ve got to be there caring. (EW, NZ European husband in his 40s, self-employed and caring for wife and 2 young children)
Products	Incontinence pads, I just, couldn’t find out from anybody, the hospice gave us what they could. I went and bought quite a few … The ones at the supermarket weren’t any good, I had to go to one of these [online companies], Nappies for Less I think it was. For the bigger ones, the larger pads. But then, once the district nurse came on board, after a few visits, I think I must have asked her, or somebody told me that they would supply them, they didn’t offer them. (CW, NZ European husband in his 60s)
Funeral/tangihanga costs	I’ve contacted the funeral directors that we dealt with with Dad and my late brother, and they’ll be the one that’s going to look after Mum when her day comes, just to get a costing … then we’ll be looking at ways of putting that cost together. And it’s not that we want her to leave us, but it’s just that we know that there’ll be a time when it comes. If we prepare ourselves now then it will make life easier for us when the time comes. (CT, Tongan daughter)
Employment	The impact for me was that I went from a salary, obviously prior to looking after mum, and gave up my job and so from a normal above average salary I went to $230 a week, so that was the impact for me. (JA, NZ European daughter)
Own health	And I thought, I can’t go through another night like this, I was just so tired and exhausted … The last four or five days are just so vivid, they were just so horrendously stressful and I was just a physical wreck. And then, of course, they die and then you’re straight into all the funeral things and I was the only one here, my brothers had to come from Australia and you’re dealing with phone calls and you’ve got deadlines for things like the paper and the funeral sheet and it just keeps going, it just keeps going. (GB, NZ European daughter, now on sleeping pills and antidepressants)
	Back injuries. (CW, NZ European husband and his adult daughter also, due to having to lift his wife in and out of bed and bathroom)

a These are illustrative verbatim quotes from interviews; in brackets are participant’s anonymised initials plus extra details to clarify context or relationships.

*Direct costs* were those involving direct outlays of money. Those most frequently mentioned were parking and transport costs related to their family member’s hospital appointments and admissions. Costs of clothing and bed linen were also mentioned by many participants; for example, some care recipients required new clothes following weight loss or required new bed linen because of increased frequency of bedding changes.

Diverse costs associated with medical treatment were reported, including those related to paying for medication and general practitioner (GP) visits and paying for alternative or complementary therapies. All participants mentioned food costs as an expenditure that had risen significantly during their family member’s illness. The final section of our results explores these costs in more detail by demonstrating how a multi-level analysis is useful in understanding the complex ways in which these costs operate. Finally, costs of funerals and tangihanga (Māori funeral customs) were discussed. Some participants’ family members had insurance to cover at least part of the cost; others reported getting estimates of costs prior to their family member’s death, so they could put financial plans in place or using government-assisted funeral subsidies. Several Māori participants anticipated funeral expenses and had taken out funeral insurance cover. Government-assisted funeral subsidies managed by Work and Income New Zealand were also utilised. Lack of resources led to several whānau being unable to conduct traditional tangihanga at ancestral homes.

*Indirect costs* were those incurred by participants as a result of their caregiving role. These included costs relating to lost employment opportunities, cultural obligations and personal costs related to carer health and well-being. Participants who were in paid work were often forced to fit in caring tasks around work, negotiating to work from home, using up annual leave and sick leave or taking unpaid leave. Some participants had to give up paid work to care; others had their benefits cut because they were unable to actively look for work because of caring responsibilities. Self-employment allowed some flexibility and also meant there were no paid leave provisions. One self-employed participant, for example, reported that his income halved when caring for his wife who had to leave her full-time job, leaving him with the ‘balancing act’ of trying to work, care for two children and care for her. After her death, he was forced to sell the family home and buy a smaller, more affordable property. For Māori, the cultural obligation and preference to return to ancestral homes before death and/or post death (tangihanga) incurred additional transport costs and other expenses associated with meeting these cultural end-of-life needs. In several cases, customary funeral traditions were interrupted due to a lack of resources. Some participants also reported that caring had negatively affected their own health and well-being. Costs incurred as a result included physiotherapy for back injuries resulting from lifting their family member. Anxiety, depression and insomnia associated with caregiving could also incur financial costs in terms of GP visits and medications, both at the time of caregiving and into bereavement.

### Palliative care context increases costs

While any type of caregiving can have financial costs associated with it, participants’ responses highlighted how the particular context of caring for someone with palliative care needs could exacerbate financial costs and constrain the mitigation of such costs. Indeed, carers had a sense of urgency about meeting the person’s needs, regardless of cost, as death approached. For example, within the Māori cohort, many carers reported purchasing kaimoana (seafood) to satisfy the cultural preferences of the dying individual, while participants in the non-Māori cohort reported sourcing very expensive food items to try and ‘tempt’ their family member to eat when appetite was affected by nausea and illness:

Mum doesn’t eat, okay? When mum feels like eating, if mum said to me, ‘Oh I feel like scallops’, I’d hunt the earth down to find scallops for Mum because if she’s going to eat that, I’d rather her have that. (ED, NZ European daughter)

Similarly, participants reported paying for anything they could that would make life easier for their family member. For example, several talked about paying extra money to receive GP home visits because of the burden of attending the practice:

We got the doctor to come and visit her […] it was just so much easier for him to come to her. Even though it cost 70 dollars instead of 15 or 20 or whatever it was. It was worth the extra costs. (BC, NZ European son)

A patient’s desire to ‘die at home’ would often come at a cost, including a financial cost, in terms of family members having to provide nursing care. For example, a Māori participant terminated paid employment to provide full-time care for her older mother, knowing that it would incur financial hardship to nurse her at home:

So I gave up my [job]; I resigned from my job which, yeah, which I knew would put me in a position where I wouldn’t be able to cope financially. (CTH, Māori daughter)

A New Zealand European son reported that his mother with bowel cancer was ‘adamant’ she wanted ‘to die at home in her own bed’ which meant that he used up all his sick and annual leave as he and his wife took on the responsibility of caring, including hourly toileting, and was ‘completely shattered’ when it was over. A man who had recently been made redundant moved in to help care for his sister, so her husband could keep working, even though he was then penalised for not actively job-seeking:

She prefers the family to care for her because obviously they know her better than any stranger from outside and it’s much better for her, so she knows who’s caring for her. Because she’d rather have family look after her than anybody else. (HH, NZ European brother)

### Costs of caring affects the wider family/network

Financial costs also had an impact on the wider family or social network, including after the death of the patient. While in most cases, the bulk of the costs were incurred by one primary carer, typically there were multiple family/whānau members involved in providing care and sharing costs.

Several Māori whānau identified they had two or more income streams flowing into their home. However, this did not always result in money being equitably dispersed to help off-set care costs. It is also important to recognise that some participants had multiple caring responsibilities, as exemplified in this daughter’s account of caring for her older parents:

Mum can’t look after Dad and Mum tries to look after Dad and then Dad tries to look after Mum and then Dad worries if Mum fell he wouldn’t be able to pick her up and so we’ve sort of taken those worries away from them both [by having Mum move in with us] … I cook meals at night for Dad and then I take them around at night for Dad and then [niece] will do everything else that needs to be doing, like the housework and the cleaning and things like that. (ED, NZ European daughter)

The complex care requirements across family networks, both in terms of time and money, could have an enduring and compounding impact on carers that many felt was not sufficiently recognised by health or social service providers, employers or wider social networks.

### Cost and care-support systems are confusing and inadequate

There were variable accounts as to what statutory (government) help was available in the context of ‘being palliative’. New Zealand has an increasingly constrained, publicly funded health system with some provision for end-of-life care, but exactly what was available, and when, was often unclear to participants. Interviewees were appreciative of help and support they received but were frustrated at systems and bureaucracies, including the apparently random discovery of entitlements, as one participant explained,

They don’t come out and give you a brochure and say, ‘Here you go, here’s an application form, fill it in and you’ll get some money’. No, you’ve got to ask so that can sometimes be extremely frustrating because you don’t know how you’re going to survive this financially. (EW, NZ European husband)

There was *not* a widespread expectation that ‘the state should provide’ or that people were entitled to a lot from the health and social system. The key issue was that if there *were* agreed provisions, it should be easy to find out about them and to use them effectively. One participant suggested that there should be a ‘palliative care needs-based assessment’, where needs were assessed across the person *and* the carer network, in order to identify resource constraints and provide clear information on costs and entitlements.

### Costs operated at a number of levels

**Figure 1.**
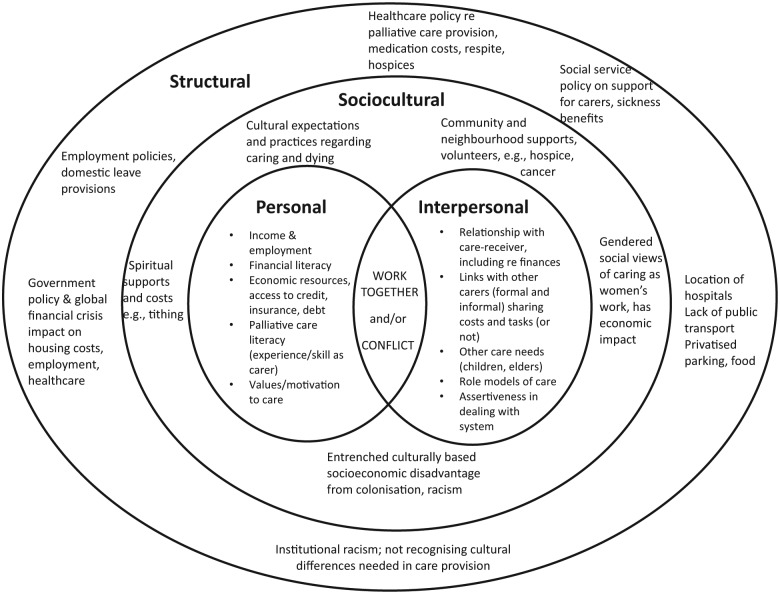
Four dimensions influence financial costs of care.

Four dimensions of costs were identified in our analyses: personal, interpersonal, sociocultural and structural.^23^ The way these dimensions interacted with one another in relation to financial costs is represented in Figure 1. Analyses identified that *personal* and *interpersonal* costs sat alongside each other and could also overlap. For example, one participant reported using up all her paid leave but being supported financially by her brothers, so she could care for their father; another, by contrast, stated that her brother offered no financial or other help but wanted money from her mother’s house sale. The central relationships between carers and care-receivers were located within the context of the *sociocultural* realm, where community or wider social or financial resources may be available, and where cultural or gendered practices around care produced particular expectations and costs. As one participant explained,

I’m the only girl, I’ve got three older brothers, they’ve all got families and other commitments. I, on the other hand (laughter) have not got any attachments at the moment, but of course being of Cook Island descent, it’s family first. (MS, Cook Island/European daughter)

Finally, the *structural* systems within which care occurs were found to have cost implications at all levels. For example, many participants appreciated New Zealand’s publicly funded health emergency services but not the lack of ongoing or palliative care provision:

I think our health system is fantastic in emergency situations and being very thorough, doing all the tests and the staff are wonderful, they are. But it’s the follow up, it’s what happens after the immediate emergency part has passed, it’s the follow up care that is inadequate … (GB, NZ European daughter)

An example of how costs operated across the four levels of the framework is outlined in Table 3 in relation to food costs. It was apparent in our analyses that food costs were related to the individual-level needs of the care-receiver to have particular foods, the interpersonal need for food to cater for visitors, the sociocultural demands for meaningful ‘cultural’ food and the structural costs of having to eat at expensive hospital cafes while remaining ‘24/7’ at the bedside of the person with a life-limiting illness.

**Table 3.** Costs of food at all levels.

Personal/individual costs	Examples
Luxury or ‘treat’ food	Pavlova, shrimp cocktail, sushi, crayfish
‘Healthy’ food	More fresh fruit and vegetables for person with illness, green milk (skim) more expensive than blue (full fat), vitamin water (helped nausea)
Effects of illness and treatment on appetite	Increased appetite (e.g. because of steroids)
	Decreased appetite/nausea: costly ‘liquid food’ or high protein drinks/meal replacements
Interpersonal costs	
Visitors	Tea/milk, biscuits for ‘daily visitors’; providing meals for many
Food for family coming to visit/care	Having to make meals for Dad now that Mum’s sick; family members coming to stay
Effect of palliative care journey on carers’ eating	Partner’s not eating, why should I? (YT); money spent on petrol/parking for hospital so less money for food bill; unhealthy hospital café food or rushed takeaway food
Minimal way to express care for sick person	Prompts siblings to at least drop off some food to Mum. (FM)
Sociocultural costs	
Food characterised as ‘cultural’	‘Island food’ – taro, yams. (KU)
	‘Food from the sea’ – sent daughter to go diving for kina; traditional food like ‘rotten corn’. (MR)
Sociocultural expectations of providing food at funeral	Couldn’t afford to cater for funeral food ‘takeaways down the road if you want it’. (YT); flat out organising everything including food for funeral when you’re exhausted. (GB); part of leftover funeral debt is cost of catering
Structural impacts on food	
Food grants	So-called ‘food grants’ (emergency support) for those on state-allocated sickness/unemployment benefits were hard to get, with narrow criteria/inflexibility re-support allocation
Hospital cafes – private businesses	Lack of healthy food options, little suitable for vegetarians/other health needs. (MS)
	High cost of food for ‘trapped’ people – 24 h/day at hospital; no other food purchase options near hospice or hospital
Poor communication systems	Not warned of discharge so no food in Mum’s house. (FM)
Hospital food systems for patients	Being sent the wrong meal or no meal – no way to know how to find hospital kitchen or request correct food. (CW)

## Discussion

This study is one of the first to explore the range, and impact, of financial costs borne by family caregivers within a palliative care context. Our findings are consistent with the limited evidence base in this area which indicates that these costs are substantial^5,9^ and can have serious and long-lasting repercussions. While significant costs have also been reported in the wider caregiving literature,^23^ it was clear that the context of a life-limiting illness was significant to the nature and extent of financial outlay.

Our findings were interpreted within the context of a multi-dimensional model of the costs of caring. Within the personal and interpersonal domains, our findings indicate that a range of direct costs are incurred by family caregivers in many diverse areas. Many of these were consistent with the previous limited research in this area, for example, the work by Dumont et al.,^24^ as well as the wider caregiving literature. Within New Zealand, in line with other countries with a partly or fully privatised health sector, the costs of unsubsidised medications and GP home visits are acknowledged to place a significant burden upon patients and family caregivers. Similarly, the costs of travelling to health appointments and parking charges, both of which were frequently raised by our participants, have also been discussed within the wider literature.^25^ Our findings confirm that large regional rather than local hospitals (advocated for economies of scale), poor public transport infrastructure (Auckland lacks good public transport) and privatised contracts for revenue-generating parking and food provision in hospitals all compounded our participants’ costs. This highlights the interplay of structural and personal/interpersonal dimensions on financial costs.

Our study also extends previous findings by identifying a range of direct costs that go beyond those related to a patient’s medical care. Food was a frequently mentioned expense, and our multi-level analysis enabled insight into the different ways in which these costs were incurred. In addition, indirect costs were reported, including those related to present and future paid employment and a carer’s own health and well-being. This finding is of particular concern given evidence which suggests that informal caregiving is often associated with poor physical and mental health, which may be exacerbated by the caring role.^26^

A range of costs was also identified within the sociocultural domain. Costs were experienced differently across the sample, with participants with limited financial means disproportionately impacted. Low-income Māori families accrued debt at a time when cultural and familial imperatives to care were at their greatest. For Māori participants, caring took place against a backdrop of economic and social disadvantage associated with the context of colonialism and disenfranchisement.^27^ The financial costs of family caregiving can therefore be seen as exacerbating pre-existing social inequities.

The structural level can also be linked to the critique of neoliberal welfare and support systems where the rhetoric that ‘family care is best’ is underpinned by a less overt assertion that ‘family care is cheapest’ (to the state).^28,29^ This echoes the use of the four dimensions in other narrative research investigating how individual narratives are inevitably intertwined with collective effects.^30^

This study was an initial exploratory project in New Zealand’s largest city, therefore findings may not be applicable to other contexts or settings. However, our study presents the first data of its kind and underlines the importance of further research to provide quantifiable data on the nature and extent of family caregiving costs.

## Conclusion

The multi-level analysis of costs offers opportunities for multi-level actions to mitigate costs from personal and family financial planning to better cultural awareness around customary practices and associated costs. The presence of statutory support for those caring for people in a palliative care context was valued; the absence of information and transparent eligibility and access to such support was not. While our findings show that financial costs were significant, there was also evidence of resourceful financial problem-solving and a strong commitment to care. However, this commitment to care cannot be realised without further research which quantifies the nature and extent of costs, the development of innovative solutions for supporting family members caring for those at the end of life and policy commitment to supporting those family caregivers who are key to palliative care provision.

## References

[1] Department of Health. End of life care strategy for England. London: Department of Health, 2008.

[2] Ministry of Health. The New Zealand palliative care strategy. Wellington, New Zealand: Ministry of Health, 2001.

[3] Department of Health. End of life care strategy: second annual report. London: Department of Health, 2010.

[4] Dunbrack J The information needs of informal caregivers involved in providing support to a critically ill loved one, http://hc-sc.gc.ca/hcs-sss/pubs/home-domicile/2005-info-caregiver-aidant/index-eng.php (2005, accessed 3 August 2014).

[5] Gardiner C Brereton L Frey R Exploring the financial impact of caring for family members receiving palliative and end-of-life care: a systematic review of the literature. Palliat Med 2004; 28(5): 375–390. 24201134 10.1177/0269216313510588

[6] Covinsky KE Goldman L Cook EF The impact of serious illness on patients’ families. SUPPORT investigators. Study to understand prognoses and preferences for outcomes and risks of treatment. JAMA 1994; 272(23): 1839–1844. 7990218 10.1001/jama.272.23.1839

[7] Jo S Brazil K Lohfeld L Caregiving at the end of life: perspectives from spousal caregivers and care recipients. Palliat Support Care 2007; 5(1): 11–17. 17461367 10.1017/s1478951507070034

[8] Covinsky KE Landefeld CS Teno J Is economic hardship on the families of the seriously ill associated with patient and surrogate care preferences? Arch Intern Med 1996; 156: 1737–1741. 8694674

[9] Chai H Guerriere DN Zagorski B The magnitude, share and determinants of unpaid care costs for home based palliative care service provision in Toronto, Canada. Health Soc Care Community 2014; 22(1): 30–39. 23758771 10.1111/hsc.12058

[10] Hughes-Hallett T Craft A Davies C Creating a fair and transparent funding system; the final report of the palliative care funding review. London: The Palliative Care Funding Review, 2011. 10.3109/15360288.2011.621020 22126166

[11] Wolcott HF Transforming qualitative data: description, analysis and interpretation. London: SAGE, 1994.

[12] Randall WL Narrative and chaos: acknowledging the novelty of lives-in-time. Interchange 2007; 38(4): 367–389.

[13] Squire C Experience-centred and culturally-oriented approaches to narrative. In: Andrews M Squire C Tamboukou M (eds) Doing narrative research. London: SAGE, 2008, pp. 41–63.

[14] Wiles JL Rosenberg MW Kearns RA Narrative analysis as a strategy for understanding interview talk in geographic research. Area 2005; 37: 89–99.

[15] Ministry of Health. The health of New Zealand adults 2011/2012: key findings of the New Zealand Health Survey. Wellington, New Zealand: Ministry of Health, 2012.

[16] Moeke-Maxwell T Nikora LW Te Awekotuku N Manaakitanga: ethical research with Māori who are dying. In: Agee M McIntosh T Culbertson P (eds) Pacific identities and well-being: cross cultural perspectives. London: Routledge, 2012, pp. 188–204.

[17] Palliative Care Council of New Zealand. National health needs assessment for palliative care phase 1 report: assessment of palliative care need. Wellington, New Zealand: Palliative Care Council of New Zealand, 2011.

[18] Patton MQ Qualitative research and evaluation methods. 3rd ed. Thousand Oaks, CA: SAGE, 2002.

[19] Lynn J Adamson JM Living well at the end of life: adapting health care to serious chronic illness in old age. Santa Monica, CA: RAND Corporation, 2003, http://www.rand.org/pubs/white_papers/WP137

[20] Health Research Council of New Zealand. Guidelines for researchers on health research involving Māori (Version 2). Auckland, New Zealand: Health Research Council of New Zealand, 2010.

[21] Johnson R Waterfield J Making words count: the value of qualitative research. Physiother Res Int 2004; 9(3): 121–131. 15560669 10.1002/pri.312

[22] Kenyon GM Randall W Narrative gerontology: an overview. In: Kenyon G Clark P de Vries B (eds) Narrative gerontology. New York: Springer, 2011, pp. 3–18.

[23] Casey B The value and costs of informal care. Paper submitted to the Commission on Funding of Care and Support in response to its call for evidence, http://www2.warwick.ac.uk/fac/soc/ier/news/casey_-_dilnot_commission_evidence_-_as_sent.pdf (accessed 19 April 2013).

[24] Dumont S Jacobs P Fassbender K Costs associated with resource utilization during the palliative phase of care: a Canadian perspective. Palliat Med 2009; 23(8): 708–717. 19837702 10.1177/0269216309346546

[25] Brooks J Wilson K Amir Z Additional financial costs borne by cancer patients: a narrative review. Eur J Oncol Nurs 2011; 15: 302–310. 21093369 10.1016/j.ejon.2010.10.005

[26] Payne S The changing profile of the family caregivers of older people: a European perspective. In: Gott M Ingleton C (eds) Living with ageing and dying: palliative and end of life care for older people. Oxford: Oxford University Press, 2011, pp. 149–157.

[27] Smith LT On tricky ground: researching the native in the age of uncertainty. In: Denzin NK Lincoln YS (eds) The SAGE handbook of qualitative research. 3rd ed. Thousand Oaks, CA: SAGE, 2005, pp. 85–107.

[28] Grenier AM Unhinging the assumptions within independence: toward a broader conceptualization of diversity and difference in home care. Can Rev Soc Pol 2003; 51: 29–47.

[29] Heaton J The gaze and visibility of the carer: a Foucauldian analysis of the discourse of informal care. Sociol Health Illness 1999; 21(6): 759–777.

[30] Cohen HL Greene RR Lee Y Older adults who overcame oppression. Fam Soc 2006; 87(1): 35–42.

